# Stage-specific ERP correlates of audiovisual facial emotion processing across depressive tendencies

**DOI:** 10.3389/fnins.2026.1810368

**Published:** 2026-04-07

**Authors:** Fengxia Wu, Ziqi Liu, Qi Dai, Zhilin Zhang, Mingwu Gu, Jingjing Yang, Lichang Yao

**Affiliations:** 1 School of Artificial Intelligence, Changchun University of Science and Technology, Changchun, China; 2 Faculty of Biomedical Engineering, Shenzhen University of Advanced Technology, Shenzhen, China; 3 Department of Neuropsychiatry, Graduate School of Medicine, Kyoto University, Kyoto, Japan; 4 Research Center for Medical Artificial Intelligence, Shenzhen Institute of Advanced Technology, Chinese Academy of Sciences, Shenzhen, Guangdong, China

**Keywords:** audiovisual facilitation, depressive tendencies, emotion processing, event-related potentials, facial expression

## Abstract

Emotional dysregulation can emerge as early as the initial stages of depression. This study aimed to examine event-related potential characteristics during the perception of negative, positive, and neutral facial expressions in healthy individuals across depressive tendencies. Twenty-six healthy participants underwent ERP measurements during emotion recognition using a facial emotion recognition task in visual and audiovisual modalities. The Emotion Regulation Questionnaire (ERQ), the Difficulties in Emotion Regulation Scale (DERS-16), and the Beck Depression Inventory II (BDI-II) assessed cognitive strategy, emotion regulation difficulties, and depression severity, respectively. Facial affect elicited larger amplitudes compared to neutral faces, from the N170 and early posterior negativity (EPN) in the temporo-occipital region to the late positive potential (LPP) in the centroparietal region. Under audiovisual conditions, P1 peak latency to negative stimuli in the temporal region exhibited significant negative correlations with DERS-16 and BDI-II scores. N170 peak latency to positive stimuli also demonstrated a significant negative correlation with BDI-II scores. Under visual conditions, EPN amplitude to negative stimuli in the occipital region exhibited a significant positive correlation with BDI-II scores. P1 and N170 latencies, or neural response speeds, and EPN amplitude, which represents emotional reaction strength, correlate with depressive tendencies in healthy individuals. These early components function as initial neural signals that may serve as electrophysiological markers of abnormal emotional processing within neuropsychological functions prior to clinical depression.

## 1 Introduction

Depression is a mental disorder characterized by persistent low mood and anhedonia. Recently, the number of individuals with depression has increased (Hidaka, 2012; Santomauro et al., 2021) and incidence is also increasing in the young (MacSweeney et al., 2025), imposing a substantial burden on families and society (Liu et al., 2020). Clinical depression diagnosis and treatment depend primarily on physician interviews and scale scores (Beck et al., 1996; Gross and John, 2003; Hamilton, 1960), and objective indicators for clinical use remain lacking. In the early disease stages, patients may exhibit abnormal endogenous emotion regulation strategies, such as reduced cognitive reappraisal ability (Bylsma, 2021; Cui et al., 2024) and increased expression inhibition (Joormann, 2010); nonetheless, current assessment methods make it difficult to reliably distinguish mild depression from situational depression (Padgaonkar et al., 2021). Additionally, depression often follows an episodic course, with symptom clustering during the acute phase and functional recovery during remission or intermittent phases (Lerner et al., 2015), which further complicates early diagnosis. Accumulating evidence suggests that emotional dysregulation is a core and early depression feature that often emerges before overt clinical symptoms, making emotion-related neural indicators promising candidates for early identification.

Emotion is a key driver of interpersonal communication and decision-making and can directly trigger physiological responses (George and Dane, 2016). Facial emotion recognition is a fundamental emotional function that enables individuals to infer others’ emotional states from facial expressions. Concurrently, it provides essential affective information that may support subsequent emotion regulation processes (e.g., enhancing positive affect or alleviating negative states), offering a useful perspective on emotion processing abnormalities. This regulatory process is particularly effective when external facial expressions align with internal regulatory intentions (Gosserand and Diefendorff, 2005; Schultheiss et al., 2008). Nevertheless, human emotional judgment does not rely solely on unimodal visual information; rather, it involves integration of multiple affective cues to construct holistic emotional perception (Choo et al., 2025; Martin et al., 2012). For example, when watching a movie, the horrific effect is significantly reduced if the sound is turned off. The brain does not process visual information in isolation but integrates multiple sensory signals, such as vision and hearing, through preliminary analysis of visual and auditory characteristics, activation of emotional response systems, retrieval of relevant memories and experiences, and integration of self-referential evaluation to form subjective experiences of “fear emotions.” Nevertheless, most facial emotion expression research relies solely on visual stimuli, and comparatively little work has examined multimodal emotions using audiovisual analysis; thus, the neural mechanisms underlying this processing require further investigation.

Facial emotion processing is a dynamic, time-dependent process. Event-related potential (ERP) research has demonstrated hierarchical temporal regulation during emotional-face processing, spanning early visual components (P1 and N170) (Bayer et al., 2012; Mareike and Annekathrin, 2014; Martini et al., 2012; Sebastian et al., 2020), the early posterior negativity (EPN) (Bruchmann et al., 2020), and the late positive potential (LPP) (Valdés-Conroy et al., 2014). P1 (80–130 ms) is among the earliest ERP components observed for facial expressions and is located in the temporal occipital region. Several studies have reported enhanced P1 amplitudes for emotional stimuli (threat-related or high-arousal) relative to neutral stimuli in both visual (Kheirkhah et al., 2021; Kroczek and Mühlberger, 2025; Zhang et al., 2013) and audiovisual modalities (Liang et al., 2022; Mori et al., 2021). Nonetheless, other studies did not observe emotion-related P1 amplitude modulation (Itier and Neath-Tavares, 2017; Mareike and Annekathrin, 2014), suggesting that emotional sensitivity at this stage may depend on experimental design, stimulus properties, or participant individual differences. Another early emotion-related ERP component is N170 (140–200 ms), which occurs in the temporal occipital region and is considered face-specific processing (Eimer et al., 2010; Hinojosa et al., 2015; Mavratzakis et al., 2016; Sebastian et al., 2020). Previous studies have found that, compared with neutral stimuli, emotional stimuli such as anger, fear, and happiness can evoke more negative N170 amplitudes (Sebastian et al., 2020). This effect is influenced by low-level visual information, such as emotional intensity (Durston and Itier, 2025; Li Y. et al., 2024; Schlossmacher et al., 2025) and by high-level tasks (Schmuck et al., 2023). EPN emerges approximately 200–300 ms following stimulus onset over occipitotemporal sites and is a robust differential negativity when contrasting emotional versus neutral facial stimuli (Bruchmann et al., 2020; Neath-Tavares and Itier, 2016). Emotionally salient information is widely interpreted as reflecting an automatic, stimulus-driven attentional bias (Kroczek and Mühlberger, 2025; Schultheiss et al., 2008; Schupp et al., 2004). Moreover, LPP is a sustained positive deflection peaking over central-parietal electrodes, starting approximately 400 ms post-stimulus onset and persisting for several 100 milliseconds (Schupp et al., 2006). Emotional faces reliably elicit larger LPP amplitudes compared to neutral faces (Durston and Itier, 2025; Kroczek and Mühlberger, 2025; Schmuck et al., 2023). Additionally, some studies indicate that emotional processing is further modulated by cross-modal valence congruency; when auditory and visual emotional cues align (e.g., an angry face paired with angry prosody), LPP amplitudes are significantly enhanced relative to incongruent pairings (Kokinous et al., 2015; Mori et al., 2021). The discrepancies across these studies regarding temporal dynamics and brain region activation confirm that facial emotion processing is not a single, localized event. Similar to affective scene processing (Vogeley et al., 2025), it involves sequential neural stages or overlapping phases that occur across distributed neural networks in distinct brain regions.

This study aimed to examine how ERP components at different stages of audiovisual facial-emotion processing relate to individual differences in emotion regulation and depressive tendencies, thereby identifying potential neurophysiological indicators of emotion regulation problems. Specifically, this study used electroencephalogram (EEG) recordings obtained while participants performed facial emotion recognition tasks and analyzed emotional expressions across processing stages (e.g., early perception and late evaluation) in visual and audiovisual modalities, respectively. The Emotion Regulation questionnaire (ERQ) assessed cognitive reappraisal (CR) and expression suppression (ES); the Difficulties in Emotion Regulation Scale (DERS-16) quantified difficult emotion regulation and related functional defects; and the second version of the Beck Depression Inventory (BDI-II) assessed depression severity. In the ERP analysis, we examined the amplitude and latency of the P1, N170, EPN, and LPP components at selected electrodes and correlated these measures with total ERQ, DERS-16, and BDI-II scores to identify characteristic neurophysiological patterns associated with emotion regulation disorders. Finally, this study aimed to integrate behavioral performance, self-evaluation scales, and ERP activity to construct a framework for facial emotion processing and to provide a theoretical basis for early identification of abnormal emotion processing.

## 2 Methods

### 2.1 Participants

The study involved 26 healthy volunteers (19 men and seven women) aged 21–27 years (mean age, 22.8 years). Based on interviews, all participants were mentally and physically healthy and had not used psychotropic substances during the 2 weeks preceding EEG recording. All participants were right-handed and had normal or corrected-to-normal vision. They were informed of the experimental procedure and voluntarily provided written informed consent.

### 2.2 Assessments

Emotion regulation strategies were assessed using the ERQ (Gross and John, 2003), which includes 10 items assessing two habitual emotion regulation strategies: CR (six items) and ES (four items). Participants rated each item on a 7-point Likert scale (1 = strongly disagree; 7 = strongly agree). Subscale scores were calculated by summing the corresponding items, with higher scores indicating greater use of each strategy. Here, CR and ES subscale scores were used in subsequent correlation analyses with the ERP measures. The Chinese version of the ERQ has been previously validated (Wang et al., 2007) and demonstrated good internal consistency (Cronbach’s *α* = 0.85 and 0.77 for CR and ES, respectively) and test–retest reliability (0.82 and 0.79, respectively).

Difficulties in emotion regulation were assessed using the DERS-16 (Bjureberg et al., 2016), which includes 16 items rated on a 5-point Likert scale (1 = almost never; 5 = almost always). Total scores were calculated by summing all items, with higher scores indicating greater overall difficulty in emotion regulation. Here, the DERS-16 total score was treated as a continuous variable and used in correlation analyses with the ERP measures. Previous validation studies of the Chinese version of the DERS-16 reported excellent internal consistency (Cronbach’s *α* = 0.95) (Xiong et al., 2025).

Depressive symptoms were assessed using BDI-II. This scale, originally developed by Beck et al. (1996) as a revision of the 1961 BDI, was used to assess depression severity. The scale includes 21 items aligned with DSM-IV diagnostic criteria for depressive disorders and assesses somatic and cognitive-affective symptoms experienced during the past 2 weeks. Items are scored on a 4-point Likert scale (0–3), and the total score is the sum of all 21 items (range, 0–63), with higher scores indicating more severe depressive symptoms. Interpretive cutoff scores are typically defined as follows: 0–13, minimal depression; 14–19, mild depression; 20–28, moderate depression; and 29–63, severe depression. This tool has been extensively validated and applied in the Chinese population. In a validation study of the Chinese version in college students, Yang et al. (2012) reported excellent psychometric properties, including a Cronbach’s α coefficient of 0.85, test–retest reliability of 0.73, and item-total correlations of 0.34–0.57, indicating high internal consistency reliability.

### 2.3 Material and procedure

The experiment included both visual and audiovisual stimuli. Visual stimuli were selected from the Karolinska directed emotion-face database (Lundqvist et al., 1998). Three facial expressions were used: happy, disgusted, and neutral, as the positive, negative, and neutral stimuli, respectively. To reduce the impact of skin color differences, convert color facial images were converted to 8-bit grayscale images using MATLAB R2025a. Auditory stimuli comprised a 1,000 Hz pure tone (100 ms duration; 5 ms rise and fall) presented through headphones. In the audiovisual condition, the tone was presented simultaneously with the visual facial stimulus.

**Figure 1:**
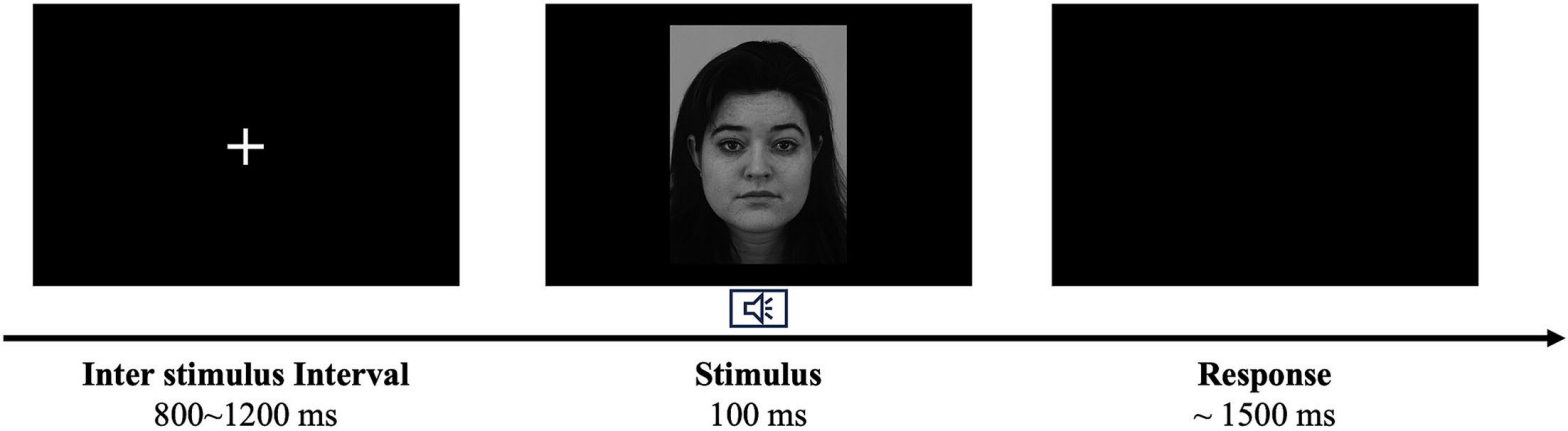
Stimulus presentation procedure.

The experiment was conducted in a quiet, soundproof laboratory at an appropriate temperature. Participants were seated 60 cm from a computer screen (resolution: 1920 × 1,080 pixels; refresh rate: 60 Hz) with their heads positioned on a chin rest. The experimental program was developed using E-Prime 2.0. As displayed in Figure 1, participants fixated on a cross on the screen during the task. Each trial began with a white cross presented at the screen center for a random duration of 800–1,200 ms. The target stimulus was then presented at the center for 100 ms, followed by a response screen for up to 1,500 ms. Participants were instructed to classify facial emotion as quickly and accurately as possible using the right hand on the keyboard (“J” for positive, “K” for neutral, and “L” for negative), regardless of whether a visual or audiovisual stimulus was presented. Before the main experiment, participants completed 24 practice trials (four trials per condition) to learn the task; only participants with a correct response rate >90% proceeded to the main experiment. The main experiment comprised six conditions, each with 60 trials (total: 360 trials), divided into two blocks. Between blocks, participants could take a break if necessary. Participants were instructed to avoid eye blinks and eye movements during stimulus presentation.

### 2.4 EEG recording and preprocessing

Continuous EEG data were recorded using a 64-channel cap with Ag/AgCl electrodes positioned according to the 10–20 system (Neuroscan) and sampled at 1,000 Hz. The Cz electrode was positioned at the midpoint between the nasal root and occipital bone and referenced to the REF electrode. Electrode impedances were maintained below 10 kΩ. Vertical and horizontal electrooculograms were recorded from electrodes placed below and lateral to the right eye. Offline EEG data were processed in MATLAB 2025a using the EEGLAB toolbox (Delorme and Makeig, 2004), and only correct trials were analyzed. Preprocessing included electrode localization, re-referencing to the average, high-pass filtering at 0.1 Hz and low-pass filtering at 30 Hz, manual removal of bad segments, interpolation of bad leads, segmentation into 800 ms epochs (−100 to 700 ms) relative to stimulus onset, and baseline correction to the 100 ms pre-stimulus interval. Independent component analysis was used to remove artifacts (e.g., eye blinks, eye movements, and channel interference). Grand-averaged data were computed for each participant, stimulus type, and electrode.

### 2.5 Statistical analysis

Accuracy and reaction times (RT) were analyzed to evaluate participant performance. Accuracy was defined as the number of correct responses divided by the number of target stimuli. To exclude unusually early or slow responses, trials with RT < 150 ms or >1,500 ms were removed. RT were calculated as the mean RT for correct responses. A 2 type (V, AV) × 3 emotion (negative, neutral, positive) repeated-measures analysis of variance (ANOVA) was performed for RT and accuracy.

Based on the average ERP results and previous literature, the ERP components analyzed were P1, N170, EPN, and LPP. Time windows were 80–160 ms, 140–230 ms, 250–330 ms, and 400–650 ms for P1, N170, EPN, and LPP, respectively. For P1 and N170, maximum amplitude and latency at occipital (O1, O2) and temporal (P7, P8) regions were extracted; for EPN, mean amplitude at occipital (O1, O2) and parietal (P7, P8) regions was selected; and for LPP, mean amplitude at centroparietal (CP1, CP2, P3, Pz, P4) regions was extracted. A 2 type (V, AV) × 3 emotion (negative, neutral, positive) repeated-measures ANOVA was conducted for each ERP component. When an interaction was significant, *post hoc* comparative analyses were performed.

Spearman’s correlation analysis was used because some scale scores demonstrated non-normal distributions. Two-tailed Spearman correlation analyses were also conducted to examine the associations between questionnaire scores and brain activity evoked by the emotion differential (positive-neutral and negative-neutral) in P1, N170, EPN, and LPP components. All analyses were performed in SPSS, with *α* = 0.05, and Bonferroni correction was applied for multiple comparisons across all behavioral and ERP analyses.

## 3 Results

### 3.1 Behavioral results

Participant accuracy and RT under each condition are displayed in Table 1. Accuracy exceeded 93% across all conditions, indicating that participants understood the task.

**Table 1:** Mean accuracy (%) and mean response times (ms).

Condition	Accuracy		Response times	
	Visual modality	Audiovisual modality	Visual modality	Audiovisual modality
Negative	93 ± 1.5	93 ± 1.5	716 ± 16	705 ± 17
Neutral	95 ± 1.5	95 ± 1.3	710 ± 12	714 ± 15
Positive	97 ± 1.0	96 ± 1.1	678 ± 14	665 ± 12

Data are expressed as mean ± standard error (SE) for all conditions.

A 2 × 3 repeated-measures ANOVA on accuracy exhibited a significant main effect of emotion, *F*(2, 50) = 8.637, *p* = 0.001, η_p_^2^ = 0.257. Accuracy for positive stimuli (96%) was significantly higher than that for neutral (95%) and negative (93%) stimuli (all *p* < 0.01). Accuracy did not differ between negative and neutral stimuli. The main effect of type and the type × emotion interaction was not significant (all *p* > 0.05).

A 2 × 3 repeated-measures ANOVA on RT showed a significant main effect of emotion, *F* (2, 50) = 10.853, p = 0.001, η_p_^2^ = 0.303. Responses to positive stimuli were significantly faster than those to negative and neutral stimuli (all *p* < 0.001). Conversely, RT did not differ between negative and neutral stimuli (*p* = 0.744). The main effect of type was also significant, *F* (1, 25) = 7.031, *p* = 0.014, η_p_^2^ = 0.220: RT for audiovisual stimuli was shorter than that for visual stimuli, indicating a crossmodal-dominance effect. The type × emotion interaction was not significant, F (2, 50) = 2.637, *p* = 0.234, η_p_^2^ = 0.257.

### 3.2 ERP results

#### 3.2.1 Latencies

##### 3.2.1.1 P1 component

In the occipital region, only the main effect of type was significant, *F* (1, 25) = 9.046, *p* = 0.006, η_p_^2^ = 0.266. P1 latency was shorter with audiovisual stimuli (144 ms) than with visual stimuli (148 ms). Further analysis indicated a crossmodal facilitation effect for positive and neutral stimuli (all *p* < 0.05). In the temporal region, the main effect of type was also significant, *F* (1, 25) = 6.329, *p* = 0.019, η_p_^2^ = 0.202: P1 latency was shorter with audiovisual stimuli (138 ms) than with visual stimuli (142 ms). Further analysis similarly demonstrated facilitation effects for positive and neutral stimuli (all *p* < 0.05). The main effect of emotion and the type × emotion interaction were not significant (all *p* > 0.05).

##### 3.2.1.2 N170 component

**Figure 2:**
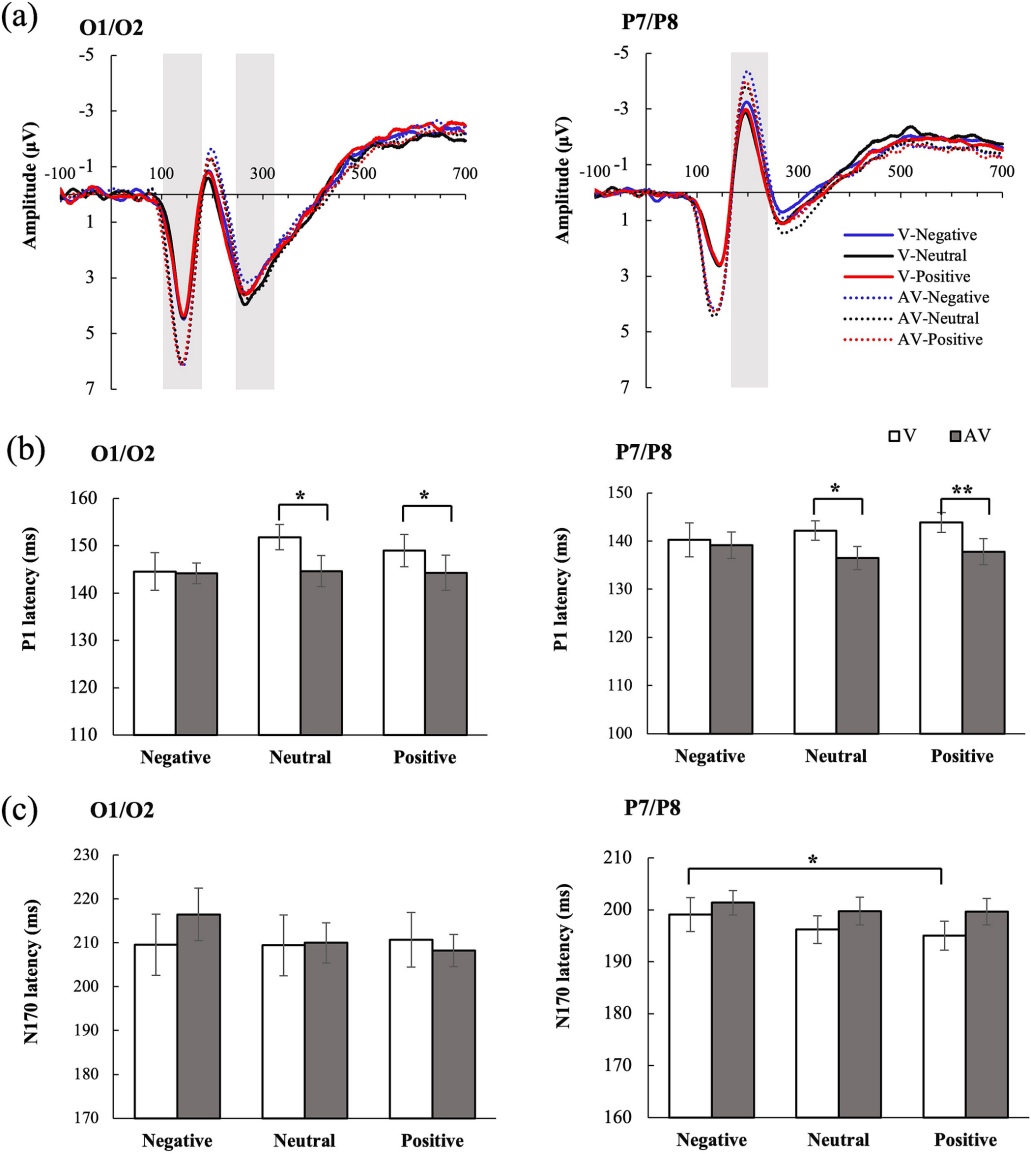
ERP waveforms and latency results. **(a)** Averaged ERPs are shown separated by visual (V) and audiovisual (AV) conditions in the occipital (left) and temporal (right) regions. **(b)** P1 latency and **(c)** N170 latency for all conditions at the occipital (left) and temporal (right) regions. Black asterisks indicate significant differences (Bonferroni-corrected): *p < 0.05, **p < 0.01, ***p < 0.001.

In the occipital and temporal regions, the main effects of emotion and type, as well as the type × emotion interaction, were not significant (all *p* > 0.05). Nonetheless, in temporal region, the N170 latency to positive stimuli (195 ms) was shorter than that to negative stimuli (199 ms) in the visual condition (see Figure 2).

#### 3.2.2 Amplitudes

##### 3.2.2.1 P1 component

In the occipital region, only the main effect of type was significant, *F* (1, 25) = 29.091, *p* < 0.001, η_p_^2^ = 0.538. The P1 amplitude for the audiovisual stimulus (7.30 uV) was significantly higher than that for the visual stimulus (5.39 uV). Further analyses indicated a crossmodal facilitation effect for all stimulus types (all *p* < 0.001).

In the temporal region, the main effect of type was significant, F (1, 25) = 302.618, *p* < 0.001, η_p_^2^ = 0.566. The P1 amplitude for the audiovisual stimulus (5.04 uV) was significantly higher than that for the visual stimulus (3.17 uV). Nevertheless, neither the main effect of emotion nor the type × emotion interaction was significant (all *p* > 0.05).

##### 3.2.2.2 N170 component

In the occipital region, only the main effect of type was significant, F (1, 25) = 10.328, *p* = 0.004, η_p_^2^ = 0.292. The N170 amplitude for the audiovisual stimulus (−2.81 uV) was more negative than that for the visual stimulus (−2.01 uV). Further analyses exhibited a facilitation effect for negative (*p* = 0.005) and neutral stimuli (*p* = 0.002). In the temporal region, the main effect of emotion was significant, *F* (2, 50) = 4.242, *p* = 0.024, η_p_^2^ = 0.145. N170 amplitudes for negative stimuli were more negative than those for neutral stimuli (*p* = 0.038). Conversely, there was no significant difference between positive and neutral stimuli (*p* = 0.547) or between positive and negative stimuli (*p* = 0.271). The main effect of type was also significant, F (1, 25) = 17.568, *p* < 0.001, η_p_^2^ = 0.413, with a more negative amplitude for the audiovisual stimulus (−4.78 uV) than for the visual stimulus (−3.77 uV). The type × emotion interaction was not significant, F (2, 50) = 0.048, *p* = 0.931, η_p_^2^ = 0.002.

##### 3.2.2.3 EPN component

**Figure 3:**
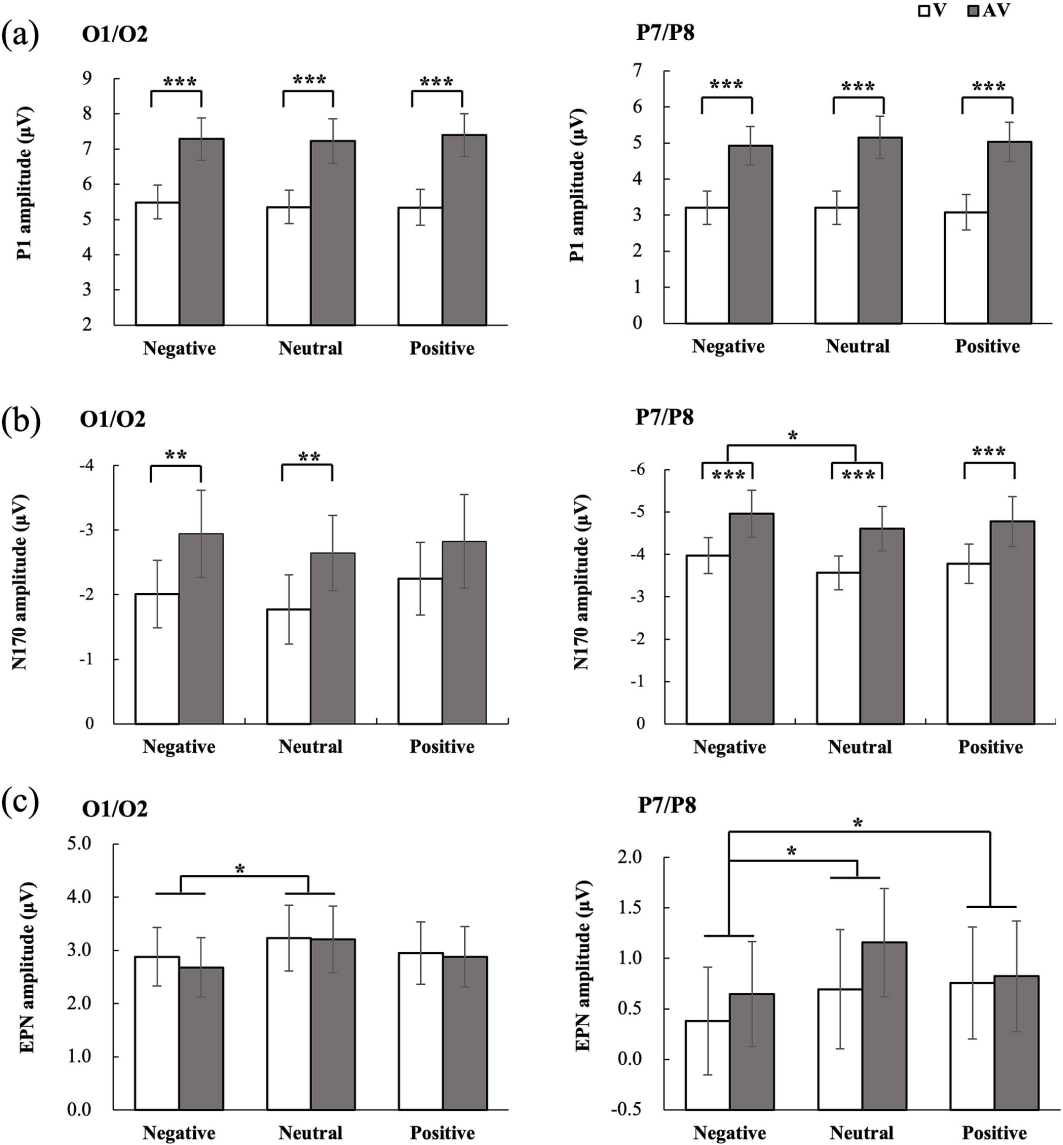
ERP amplitude results. The amplitude of **(a)** P1, **(b)** N170, and **(c)** EPN components for visual (V) and audiovisual (AV) conditions at the occipital (left) and temporal (right) regions. Black asterisks indicate significant differences (Bonferroni-corrected): *p < 0.05, **p < 0.01, ***p < 0.001.

In the occipital region, only the main effect of emotion was significant, F (2, 50) = 4.838, *p* = 0.020, η_p_^2^ = 0.162. Amplitudes for negative (*p* = 0.029) were more negative than those for neutral stimuli; nevertheless, there was no significant difference between positive and negative stimuli (*p* = 0.574). In the temporal region, a significant main effect of emotion was observed, F (2, 50) = 5.863, *p* = 0.007, η_p_^2^ = 0.190. Amplitudes for negative stimuli were more negative than those for neutral (*p* = 0.019) and positive stimuli (*p* = 0.027), whereas there was no significant difference between positive and neutral stimuli (*p* = 0.905). However, neither the main effect of type nor the type × emotion interaction was significant (*p* > 0.05) (see Figure 3).

##### 3.2.2.4 LPP component

**Figure 4:**
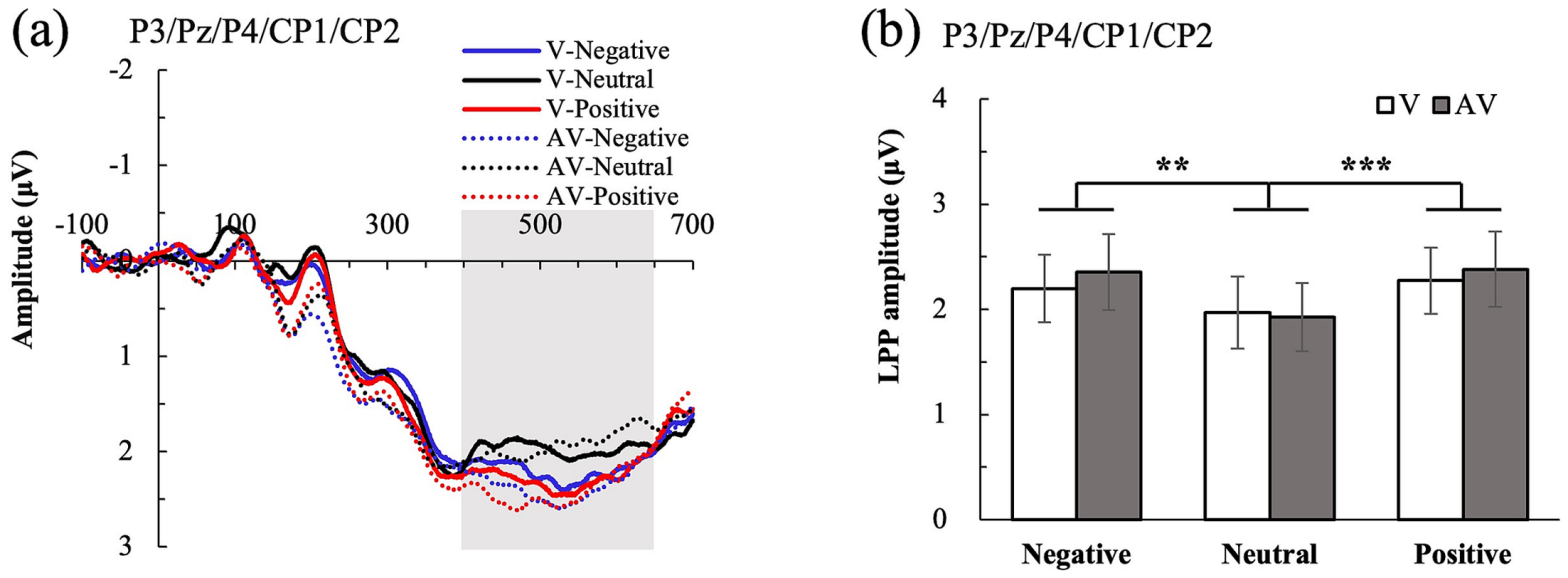
LPP component. **(a)** Averaged ERPs are shown separated by visual (V) and audiovisual (AV) conditions in the centro-parietal region. **(b)** The amplitude of LPP component for all conditions. Black asterisks indicate significant differences (Bonferroni-corrected): *p < 0.05, **p < 0.01, ***p < 0.001.

In the centro-parietal region, only the main effect of emotion was significant, F (2, 50) = 13.499, *p* < 0.001, η_p_^2^ = 0.351. LPP amplitudes for negative and positive stimuli were larger than those for neutral stimuli (all *p* < 0.01); nonetheless, there was no significant difference between positive and negative stimuli (*p* = 0.792). There were no significant main effects of type and no type × emotion interaction (all *p* > 0.05) (see Figure 4).

#### 3.2.3 Correlation

**Figure 5:**
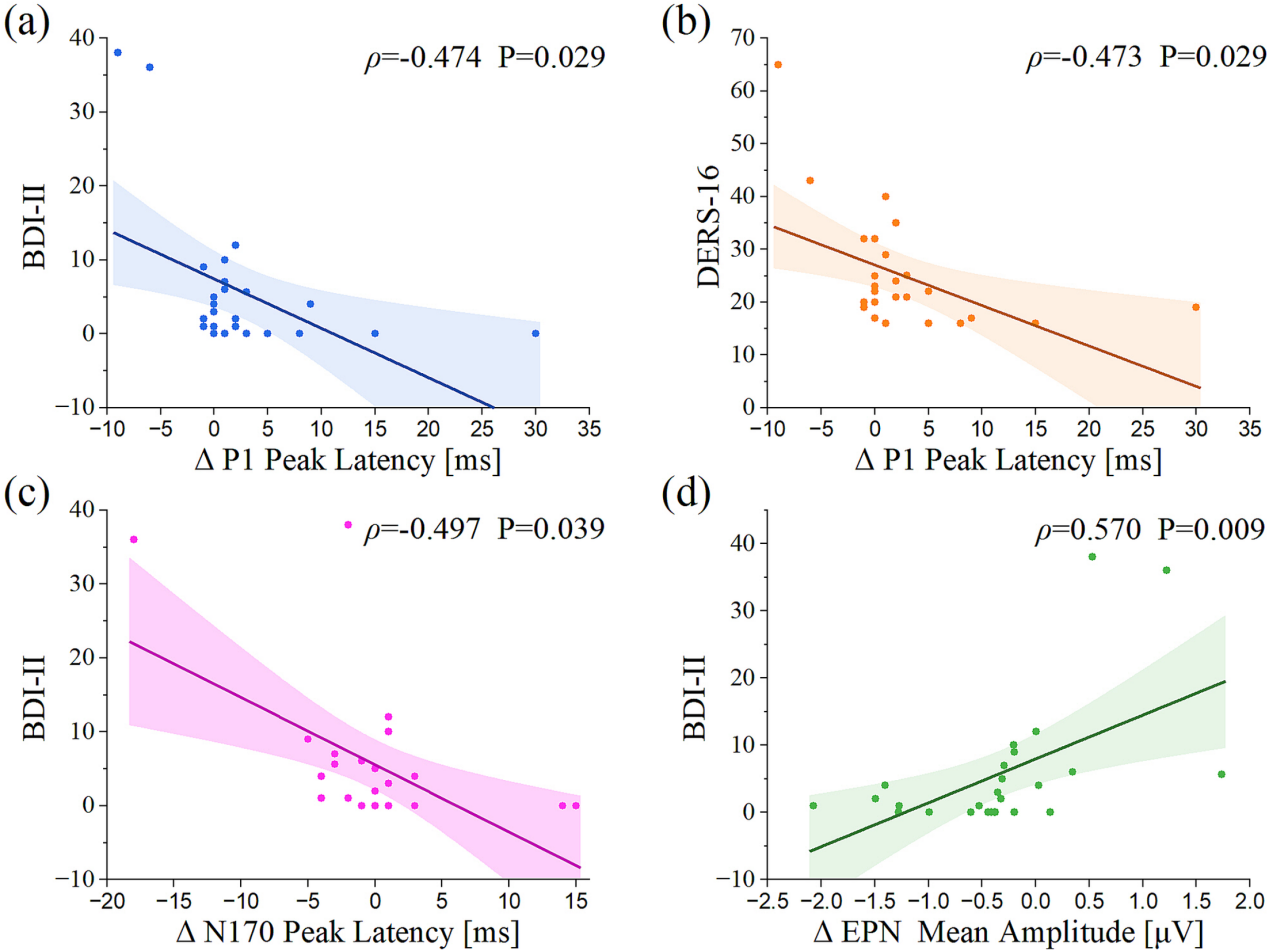
Correlation of ERP components with psychological assessments. **(a)** Correlation between BDI-II and P1 latency elicited by the audiovisual negative-neutral condition. **(b)** Correlation between DERS-16 and P1 latency elicited by the audiovisual negative-neutral condition. **(c)** Correlation between BDI-II and N170 latency elicited by the audiovisual positive-neutral condition. **(d)** Correlation between BDI-II and EPN amplitude elicited by the visual negative-neutral condition. *ρ*, Spearman correlation coefficient; *P*, probability value. Δ denotes the subtraction of neutral stimuli values from negative or positive stimuli values within the visual or audiovisual conditions.

The mean scores and standard deviations for each scale are presented in Table 2. Table 2 also reports the correlations among ERQ-ES, ERQ-CR, DERS-16, and BDI-II. The BDI-II score was positively correlated with the DERS-16 score (*ρ* = 0.709, *p* < 0.001) and negatively correlated with the ERQ-CR score (*ρ* = −0.465, *p* = 0.017). Additionally, the DERS-16 score was negatively associated with the ERQ-CR score (*ρ* = −0.418, *p* = 0.033). Correlation analysis exhibited significant associations between specific ERP components and depression severity (BDI-II) and difficulty in emotion regulation (DERS-16), as displayed in Figure 5. Under the audiovisual condition, P1 peak latency in the temporal region for the negative-neutral condition was negatively correlated with DERS-16 and BDI-II scores (*ρ* = −0.473, *p* = 0.029; *ρ* = −0.474, *p* = 0.029, respectively). N170 peak latency for the positive-neutral condition was also negatively correlated with BDI-II scores (*ρ* = −0.497, *p* = 0.039). Under the visual condition, EPN amplitude in the occipital region for the negative-neutral condition was positively correlated with BDI-II scores (*ρ* = 0.570, *p* = 0.009). However, LPP amplitude in the centroparietal regions was not correlated with scale scores.

**Table 2:** Scores and correlation with the scales.

Correlation	M ± SD	BDI-II	ERQ-CR	ERQ-ES	DERS-16
BDI-II	5.64 ± 9.86	1			
ERQ-CR	33.12 ± 6.74	−0.465*	1		
ERQ-ES	12.56 ± 4.54	0.058	0.211	1	
DERS_16	25.04 ± 11.09	0.709***	−0.418*	−0.147	1

BDI-II, Beck Depression Inventory–Second Edition; DERS-16, Difficulties in Emotion Regulation Scale (16-item version); ERQ-CR, Emotion Regulation Questionnaire–Cognitive Reappraisal; ERQ-ES, Emotion Regulation Questionnaire–Expressive Suppression; M, mean; SD, standard deviation; ρ, Spearman correlation coefficient. Black asterisks indicate significant differences (Bonferroni-corrected): *p < 0.05, **p < 0.01, ***p < 0.001.

## 4 Discussion

This study examined the association between subclinical depressive tendencies and neural processing of emotional faces in a healthy population by integrating ERP measures with standardized psychological assessments. The main findings indicate that higher depressive tendencies are significantly associated with multiple early-stage emotion-processing indices: early attentional orientation to negative stimuli (P1 latency), structural coding efficiency for positive stimuli (N170 latency), and automated selective attention to negative stimuli (EPN amplitude). Therefore, altered neural processing related to emotion regulation may precede overt clinical depression, providing electrophysiological evidence relevant to neural mechanisms underlying depression risk.

Our work has linked the degree of depressive tendency with the P1 component during emotional face processing. In the audiovisual negative-neutral condition, both the BDI-II score (Figure 5a) and the DERS score (Figure 5b) demonstrated a significant negative correlation with P1 latency. These results indicate that the higher depressive tendency levels are associated with poorer emotional regulation abilities. This pattern aligns with prior evidence that individuals with elevated depressive tendencies often exhibit impaired emotion regulation (Dickey et al., 2024; Li et al., 2019). The correlation analysis between the scales in this study also confirms this interpretation, given the significant positive correlation between BDI-II and DERS scores, see Table 2. P1 is an early visual perceptual component reflecting early visual attention and low-level stimulus features (Bruchmann et al., 2020; Liang et al., 2022; Sebastian et al., 2020). In this study, the latency of P1 for negative stimuli was shortened, which may reflect an early attentional bias or a heightened perceptual sensitivity towards negative stimuli related to early symptoms of depression. The studies by Ruohonen et al. (2020) and Kratochwil et al. (2025) support this interpretation. Ruohonen et al. (2020) reported that, in a depression patient group, negative emotional faces elicited larger P1 amplitudes compared to neutral faces, even when faces were task-irrelevant (Kratochwil et al., 2025). In contrast, this difference was not observed in a healthy control group, indicating a heightened sensory receptivity toward negative emotional information in depression. Similarly, Kratochwil et al. (2025) found that the right brain dominance in emotional processing was weakened in patients with major depression, further suggesting altered early neural processing patterns. Notably, the present study identified a correlation between P1 latency and depression tendency in healthy individuals, suggesting that neural features of emotional-processing alterations may be detectable before clinical depression onset.

Moreover, in the audiovisual positive-neutral condition, the BDI-II score was negatively associated with the N170 latency (Figure 5c), indicating that shorter N170 latencies to positive stimuli were associated with higher depressive tendency. Depression is typically characterized by a negative emotional bias (Wu et al., 2016; Zhao et al., 2015) and a blunted response to positive stimuli (Chen et al., 2014; Tong et al., 2020; Xi et al., 2023). Accordingly, prior studies reported prolonged N170 latencies to positive emotional faces in patients with depression (Chen et al., 2014; Tong et al., 2020). Further, Xi et al. (2023) reported a positive correlation between N170 latency and Dysfunctional Attitude Scale scores, a measure of depressive cognitive schemas, although they only used neutral stimuli. Conversely, the present study did not show a blunted positivity effect despite using happy facial expressions as positive stimuli. Instead, the response pattern for positive stimuli resembled that for negative stimuli. This discrepancy may reflect evidence that the N170 component is more sensitive to emotional arousal than to stimulus valence (Hinojosa et al., 2015; Schindler et al., 2023).

Furthermore, the EPN component amplitude was related to depressive tendency. In the visual negative-neutral condition, EPN amplitude exhibited a significant positive correlation with BDI-II scores (Figure 5d), suggesting that higher depressive tendency was associated with enhanced neural responsiveness to negative stimuli. EPN is a well-established ERP component of early automatic affective processing (Schmuck et al., 2023), characterized by relative negativity over posterior scalp sites approximately 200–300 ms after stimulus onset. Convergent evidence identifies EPN as a marker of automated selective attention to motivationally salient stimuli, typically quantified as a differential response (emotional minus neutral) within this latency window (Langeslag and van Strien, 2018). Accordingly, previous studies reported larger (i.e., more negative) EPN amplitudes for fearful (Bruchmann et al., 2020), disgust (Wheaton et al., 2013), angry (Kroczek and Mühlberger, 2025), as also happy (Durston and Itier, 2025) stimuli than for neutral stimuli. From an evolutionary perspective, amplified EPN responses to threat-related cues may support rapid environmental monitoring and behavioral preparedness (Li S. et al., 2024). Importantly, aberrant EPN modulation has been implicated in affective disorders; for example, individuals with anxiety disorders demonstrate heightened EPN amplitudes to fearful faces, which supports an early attentional bias toward negative information (Yoon et al., 2016). Furthermore, the present study found a positive association between EPN magnitude and depressive tendency even in healthy individuals, suggesting that emotional processing disorders may precede clinical depression symptoms and that EPN may serve as an electrophysiological indicator for early depression identification.

In this study, we also examined the relationship between LPP amplitude and psychometric scores; however, our results revealed no correlation between centroparietal LPP activity and these scores. This finding may be associated with the progression of depressive disorder (Du et al., 2024; McGhie et al., 2021; Santopetro et al., 2021; Zheng et al., 2024). Consistent with our results, McGhie et al. (2021) reported that in healthy female adults, the difference in LPP amplitude between emotional and neutral stimuli was not correlated with depression subscale scores—even though pleasant and unpleasant emotional stimuli elicited significantly larger LPP amplitudes than neutral stimuli (McGhie et al., 2021). Similarly, Du et al. (2024) observed that individuals with subthreshold depression exhibited reduced N100 amplitudes but not LPP differences when processing positive facial expressions. For patients with clinical depression, Santopetro et al. (2021) demonstrated a significant negative correlation between the mean centroparietal LPP amplitude (300–600 ms) and BDI-II scores. Furthermore, Zheng et al. (2024) found that patients with major depressive disorder (MDD) showed significant cognitive impairment in the centroparietal region at 300–500 ms, and that the MDD group exhibited larger P3 difference amplitudes than the healthy control group in both non-emotional and emotional tasks. These results suggest that depressive trait vulnerability is more strongly reflected in early automatic processing rather than in later evaluative stages. In addition, the current study utilized an active emotion classification task in which participants performed overt motor responses. As such, late ERP activity (e.g., the LPP) may partly reflect motor preparation processes, a factor that may also have influenced the observed correlations. For instance, Zhang et al. (2021) used an oddball paradigm to investigate differences in implicit emotional processing between individuals with subthreshold depression and healthy controls; they reported a significantly smaller centroparietal P3 peak amplitude in the subthreshold depression group, as well as a positive correlation between this amplitude and depression scale scores. It is therefore plausible that significant associations were observed for earlier ERP components rather than the LPP within our healthy collegiate sample.

Critically, auditory input modulated the association between neural responses during facial emotion processing and depressive tendency. Correlations between P1, N170 latencies and depressive tendency were present in the audiovisual modality but not in the visual-only modality. In the audiovisual modality, we observed increased P1 and N170 component amplitudes for all emotional stimuli. The current findings are consistent with prior studies (Liang et al., 2022; Wu et al., 2025), suggesting a more robust sensory processing due to audiovisual facilitation. In addition, the shorter P1 latencies observed for audiovisual (relative to visual) positive and neutral stimuli suggest that the accompanying meaningless sounds influenced facial emotion processing and facilitated audiovisual response. Overall, the sound boosted the audiovisual integration by bottom-up processing in early perceptual stage. However, in the present study, only non-emotional pure sounds were used, and future research should manipulate auditory emotional valence (consistency vs. conflict) to further examine cross-modal integration effects.

## 5 Limitations

Regarding the findings of the present study, several limitations should be acknowledged. First, the sample size was relatively small, and a sex imbalance was evident: the sample comprised 26 healthy undergraduate students, with 19 males and seven females. Second, the relatively large standard deviation of the BDI-II scores in this study was due to the high scores of two participants. However, both participants strictly met the recruitment criteria for healthy volunteers (college students with no clinical mental illness). Furthermore, their scores were not outliers across all measures; for example, their scores on the ERQ scale remained within the normal range. In addition, we used Spearman correlation, which is more robust to outliers, and applied strict adjusted *p*-value correction. Although the findings might be slightly affected by individuals with relatively high depressive tendencies, these participants were retained in the correlation analysis. Third, we analyzed the full 0.1–30 Hz frequency band as a whole, rather than conducting separate analyses for distinct sub-bands. Finally, an active emotion classification task was employed in the study, meaning that the LPP may partly reflect motor preparation processes, rather than purely emotion-related neural processing. Future studies should adopt a passive viewing paradigm to further investigate the relationship between depressive tendencies and late evaluative emotional processing.

## 6 Conclusion

In summary, the present study examined the association between subclinical depressive tendencies and neural processing of emotional faces in a healthy population using visual and audiovisual modalities. Prior work has confirmed cross-modal specificity in early neural activity during facial emotion processing. Additionally, P1 latency and EPN amplitude for negative stimuli and N170 latency for positive stimuli were significantly correlated with an individual’s degree of depression tendencies. These results provide direct electrophysiological evidence that individuals at risk for depression show emotional processing bias at the early perception stage.

## Data Availability

The original contributions presented in this study are included in this article/supplementary material, further inquiries can be directed to the corresponding authors. Requests to access the datasets should be directed to wufengxia@cust.edu.cn.
